# Potentially Zoonotic Viruses in Wild Rodents, United Arab Emirates, 2019—A Pilot Study

**DOI:** 10.3390/v15030695

**Published:** 2023-03-07

**Authors:** Pia Weidinger, Jolanta Kolodziejek, Tamer Khafaga, Tom Loney, Brigitte Howarth, Moayyed Sher Shah, Ahmad Abou Tayoun, Alawi Alsheikh-Ali, Jeremy V. Camp, Norbert Nowotny

**Affiliations:** 1 Viral Zoonoses, Emerging and Vector-Borne Infections Group, Institute of Virology, University of Veterinary Medicine Vienna, 1210 Vienna, Austria; 2 Dubai Desert Conservation Reserve, Emirates Group, Dubai P.O. Box 686, United Arab Emirates; 3 College of Medicine, Mohammed Bin Rashid University of Medicine and Health Sciences, Dubai P.O. Box 505055, United Arab Emirates; 4 American University in Dubai, Al Sufouh 2, Dubai P.O. Box 28282, United Arab Emirates; 5 Al Jalila Genomics Center of Excellence, Al Jalila Children’s Specialty Hospital, Dubai 7662, United Arab Emirates; 6 Center for Genomic Discovery, Mohammed Bin Rashid University of Medicine and Health Sciences, Dubai P.O. Box 505055, United Arab Emirates; 7 Dubai Health Authority, Dubai P.O. Box 4545, United Arab Emirates; 8 Center for Virology, Medical University of Vienna, 1090 Vienna, Austria

**Keywords:** Gerbil, *Gerbillus*, UAE, MERS-CoV, CCHFV, AHFV, RusV, hantavirus, poxvirus, herpesvirus

## Abstract

The majority of emerging viral infectious diseases in humans originate from wildlife reservoirs, such as rodents and bats. We investigated a possible reservoir, namely wild gerbils and mice trapped in a desert reserve within the emirate of Dubai, United Arab Emirates (UAE). In total, 52 gerbils and 1 jird (Gerbillinae), 10 house mice (*Mus musculus*), and 1 Arabian spiny mouse (*Acomys dimidiatus*) were sampled. Oro-pharyngeal swabs, fecal samples, attached ticks, and organ samples (where available) were screened by (RT-q)PCR for the following viruses: Middle East respiratory syndrome-related coronavirus, Crimean-Congo hemorrhagic fever orthonairovirus, Alkhumra hemorrhagic fever virus, hantaviruses, Lymphocytic choriomeningitis mammarenavirus, Rustrela virus, poxviruses, flaviviruses, and herpesviruses. All of the samples were negative for all investigated viruses, except for herpesviruses: 19 gerbils (35.8%) and seven house mice (70.0%) were positive. The resulting sequences were only partly identical to sequences in GenBank. Phylogenetic analysis revealed three novel betaherpesviruses and four novel gammaherpesviruses. Interestingly, species identification of the positive gerbils resulted in eight individuals clustering in a separate clade, most closely related to *Dipodillus campestris*, the North African gerbil, indicating either the expansion of the geographic range of this species, or the existence of a closely related, yet undiscovered species in the UAE. In conclusion, we could not find evidence of persistence or shedding of potentially zoonotic viruses in the investigated rodent cohorts of limited sample size.

## 1. Introduction

Rodentia are the largest order of mammals, consisting of more than 2200 species, representing about 43% of all mammalian species. Several rodents are well-known carriers of viruses including zoonotic viruses [1]. In the present study, we tested rodents in a hitherto under-investigated environment, namely a desert area within the United Arab Emirates (UAE), for the presence of (zoonotic) viruses. Viral zoonotic diseases that are found on the Arabian Peninsula include Middle East respiratory syndrome (MERS), Crimean-Congo hemorrhagic fever (CCHF), and Alkhumra hemorrhagic fever (AHF) [2].

MERS: Dromedary camels (*Camelus dromedarius*) have been identified as the primary source of human infection with Middle East respiratory syndrome-related coronavirus (MERS-CoV, a *Betacoronavirus*) [3,4]. They seem to be, despite rare spill-over events to other animal species [5], the only natural host of MERS-CoV, and transmission between camels likely occurs via the respiratory route. Since essentially all older dromedaries exhibit neutralizing antibodies to MERS-CoV [3], the virus seems to be ubiquitous. Investigations of wild rodent species for the presence of MERS-CoV have not been carried out so far. However, it is not unlikely that rodents may be involved in the transmission of MERS-CoV between dromedaries, either via fecal–oral or respiratory routes via aerosolized excreta.

CCHF: Crimean-Congo hemorrhagic fever orthonairovirus (CCHFV; family *Nairoviridae*) is a tick-borne, zoonotic virus that is found across Africa, Asia, Europe, and the Middle East [2,6,7,8]. Transmission cycles involve *Hyalomma* ticks and mammalian livestock, such as cattle, sheep, and goats. While humans are usually infected by exposure to contaminated blood, bodily fluids, or animal tissues of viremic animals at abattoirs or private slaughter, infections may also occur through tick bites as well as by nosocomial transmission [6,7,9]. In most animals, the infection remains asymptomatic [2], however, the disease can be severe and even fatal in humans, with a case fatality ratio (CFR) of up to 40% [6,7,9]. Small vertebrates including rodents are involved in the life cycle of certain *Hyalomma* tick species and thus in the transmission of CCHFV [8].

AHF: In 1995, a novel flavivirus (family *Flaviviridae*) causing hemorrhagic fever was isolated in Saudi Arabia. It was named Alkhumra hemorrhagic fever virus (AHFV) after the Alkhumra district in Jeddah, where the first cases were described. Of note, in some publications, the virus was mistakenly named ‘Alkhurma’ virus due to typographical error [10]. Human cases of AHF on the Arabian Peninsula have mainly been linked to sheep and camels, even though AHFV has never been isolated from livestock animals. However, it has been found in the camel tick *Hyalomma dromedarii* in Saudi Arabia, Yemen, and Kuwait [2,11]. The role of camels, sheep, goats, and other mammals including rodents regarding the maintenance and propagation of the virus remains unclear. AHFV may be transmitted to humans by direct contact to infected livestock, their raw meat, consumption of raw unpasteurized camel milk, or possibly by mosquito or tick bites [10,12].

In addition to the viruses that were described above that are known to be endemic in the UAE, we screened our samples for hantaviruses, lymphocytic choriomeningitis virus, Rustrela virus, poxviruses, and flaviviruses. These are all zoonotic viruses, or virus taxa that include zoonotic viruses, harbored by various rodent species in other parts of the world. The presence and/or abundance of many of these viruses has not yet been established on the Arabian Peninsula, although it is possible that they are present but have not yet been detected. Finally, we analyzed the samples for herpesviruses to increase our knowledge on the genetic diversity of herpesviruses in wild rodents on the Arabian Peninsula.

Hantaviruses: Each of the approximately 40 well-described orthohantaviruses at present are closely associated with one single rodent or insectivore host, and it is generally accepted that these pairs have co-evolved. Therefore, hantaviruses do not cause apparent clinical disease in their primary hosts [13]. Humans usually get infected through inhalation of viral particles from dried up feces or saliva of persistently infected rodents, resulting in asymptomatic infection or overt clinical disease [14]. Hemorrhagic fever with renal syndrome and hantavirus cardiopulmonary syndrome are the major clinical manifestations of hantavirus infections in humans. Hantaviruses continue to pose a significant public health issue. In recent years, approximately 200,000 people have been infected worldwide every year [14].

LCMV: Lymphocytic choriomeningitis mammarenavirus (LCMV) belongs to the family *Arenaviridae* and is found across the world because of its primary host reservoir, the house mouse (*Mus musculus*). In immunocompetent humans, LCMV infection is usually asymptomatic, but may cause febrile illness and aseptic meningitis. When the virus spills over to immunocompromised persons, it can lead to systemic infection and death. In addition, severe congenital disease has been reported as a result of LCMV infection. Besides its primary host reservoir, the virus has also been described in other species of rodents [15].

Rustrela virus: Rustrela virus (RusV, Rubivirus strelense, family *Matonaviridae*) was first identified in 2020 in the brain tissues of acutely ill zoo animals in Germany (a donkey, a capybara, and a red-necked wallaby), all of which succumbed to the disease. Subsequently, it was also found in yellow-necked field mice (*Apodemus flavicollis*) in and around the zoo area [16]. RusV is a relative of rubella virus, the etiologic agent of rubella in humans [16]. Recently, RusV has been identified as the causative agent of a neurological disorder called ‘staggering disease’ in domestic cats (*Felis catus*) [17], first described in Sweden in the 1970s and in Austria in the 1990s [18,19]. Furthermore, RusV was detected in wood mice (*Apodemus sylvaticus*) from Sweden [17]. Since this virus was identified only three years ago [16], nothing is known so far about further reservoir species and its geographic distribution.

Poxviruses: Poxviruses recently came into focus with the 2022 mpox (former name: monkeypox) multi-country outbreak [20]. Both orthopox- (mainly camelpox) and parapoxvirus infections have been demonstrated in the UAE [21,22]. While for certain orthopoxvirus species, such as cowpox [23,24] or mpox viruses, rodents serve as reservoir hosts, this is not the case for parapoxviruses. Thus, for our investigations we used PCR assays which detect orthopoxvirus DNA rather than parapoxvirus nucleic acid.

Flaviviruses: The genus *Flavivirus* is comprised of over 70 different viruses, many of which are important human pathogens, such as dengue viruses, West Nile virus, yellow fever virus, Japanese encephalitis virus, St. Louis encephalitis virus, tick-borne encephalitis virus [25], and the aforementioned Alkhumra virus. West Nile virus, usually transmitted by mosquitoes, but occasionally also by ticks [26], extended its geographic range to the Americas and to Europe [27,28], but is also present on the Arabian Peninsula including the UAE [29]. Other flaviviruses reported in the UAE include Bagaza and Barkedji viruses [30].

Herpesviruses: Approximately 120 different members of the family *Herpesviridae* have been detected hitherto in a vast variety of species, which presumably represents only a small fraction of the herpesviruses that actually exist. Similar to hantaviruses, each strain is closely associated with its respective primary host species, indicating that herpesviruses and their hosts have co-evolved over long periods of time [31]. Herpesviruses establish latency in their primary host species. While infections with alphaherpesviruses often lead to acute diseases, infections with beta- and gammaherpesviruses usually remain asymptomatic, at least in immunocompetent hosts.

The majority of emerging infectious diseases originate from zoonotic reservoirs [32,33], such as rodents and bats, which together constitute two thirds of all mammalian species [1,34]. Thus, we investigated members of the order Rodentia that were collected at the Dubai Desert Conservation Reserve (DDCR) in the emirate of Dubai (UAE) for a broad spectrum of possible viruses they may carry. Typical wild rodent species of the family Muridae found within DDCR are the Cheesman’s gerbil (*Gerbillus cheesmani*), the Balochistan gerbil (*Gerbillus nanus*), the Sundevall’s jird (*Meriones crassus*), and the Arabian spiny mouse (*Acomys dimidiatus*) [35,36].

## 2. Materials and Methods

### 2.1. Trapping and Sampling

In total, 52 gerbils and one jird (Gerbillinae), 10 house mice, and one Arabian spiny mouse were caught and sampled at the DDCR from March through May 2019, from various habitats such as gravel plains, sand dunes, and rock outcrops. A total of 40 gerbils were morphologically determined as *Gerbillus cheesmani*, 10 as *Gerbillus nanus*, and the jird as *Meriones crassus*. There were two gerbils that could not be identified. Apart from the house mice, all rodents were trapped in their natural habitats overnight using Sherman live traps, sampled the next morning and immediately released thereafter. One gerbil succumbed to injuries that were acquired prior to trapping. A total of 40–50 traps were laid out per trapping night in a transect line spaced 20 m apart. All house mice were trapped in the course of pest control measures at the DDCR animal food store, using classical mousetraps and collected as carcasses. From the 53 trapped Gerbillinae, 48 oro-pharyngeal swabs, 51 fecal samples, as well as 28 ticks from 18 animals were collected. Swabs, feces, and ticks were collected in tubes, intact mice in plastic bags, and all samples were stored at −80 °C at the laboratory of the College of Medicine, Mohammed Bin Rashid University of Medicine and Health Sciences, Dubai, UAE, before shipment on dry ice to the University of Veterinary Medicine Vienna, Austria, for pathological and virological investigations.

### 2.2. Pathological Investigations

Carcasses were initially investigated pathologically, and after dissection their inner organs were pooled (brain, heart, lung, liver, kidney, and spleen).

### 2.3. Preparation and Nucleic Acid Extraction of Ticks, Organ Samples, Oro-Pharyngeal Swabs, and Fecal Specimens

Adult ticks were identified morphologically on an ice-cold plate using various identification keys; 21 were nymphs, two larvae, and five adult female *Hyalomma dromedarii*. Adult frozen ticks were divided with a parasagittal section using a sterile scalpel, and one half of each was transferred to buffered saline. A total of 300–500 µL of a virus inactivation solution (DNA/RNA Shield; ZymoResearch, Irvine, CA, USA) was added to each sample, depending on the sample quantity. Feces, organ, and tick samples were homogenized using two 2.8 mm ceramic beads (Bertin Technologies, Montigny-le-Bretonneux, France) and a TissueLyser II (QIAGEN, Hilden, Germany), and then frozen at −80 °C for at least one hour. All the samples were subsequently thawed, vortexed, and centrifuged for 2 min at 6200× *g*. Automated total nucleic acid extraction was performed using 200 µL of each supernatant and QIAamp 96 Virus QIAcube HT Kit on a QIAcube HT device (plate format), or 150 µL and QIAamp Viral RNA Mini QIAcube Kit on a QIAcube machine (for 12 samples; all from QIAGEN), according to the manufacturer’s instructions.

### 2.4. Screening of the Extracted Samples for Viral Nucleic Acids

All extracts were screened by (reverse transcription real-time) polymerase chain reaction [(RT-q)PCR] for the presence of the following viruses: MERS-CoV, CCHFV, AHFV, hantaviruses, LCMV, RusV, poxviruses, flaviviruses, and herpesviruses (except for the tick samples). All PCR conditions, kits, primers, and probes that were used are shown in Table 1. All (RT-q)PCRs included negative and positive controls, and are in routine use in our laboratory as internally- and externally-validated diagnostic tests.

For CCHFV RT-qPCR, RealStar CCHFV RT-PCR Kit 1.0 was used according to the manufacturer’s instructions (altona Diagnostics, Hamburg, Germany). All other RT-qPCRs were performed using Quantabio qScript XLT 1-Step RT-qPCR ToughMix (Quantabio, Beverly, MA, USA) with primers and probes at concentrations of 0.5 µM each and the following conditions: 50 °C for 15 min, 95 °C for 2 min, and 45 cycles of 95 °C for 15 s, and 60 °C for 30 s. All RT-qPCRs were carried out on the Applied Biosystems 7500 Real-Time PCR system (Thermo Fisher Scientific, Waltham, MA, USA) or qTOWER³ G (Analytik Jena, Jena, Germany). Conventional RT-PCRs were performed using QIAGEN OneStep RT-PCR Kit (QIAGEN) under the following conditions (if not stated otherwise in Table 1): 50 °C for 30 min, 95 °C for 15 min, 50 cycles of 94 °C for 30 s, 60 °C for 30 s, and 72 °C for 30 s, and a final elongation step at 72 °C for 7 min. Conventional PCRs were performed using QIAGEN Fast Cycling PCR Kit (QIAGEN), under the conditions stated in Table 1.

### 2.5. Molecular Rodent Species Identification

In addition, several PCRs were performed in order to confirm the species identities of the rodents, the ones resulting in specific bands are shown in Table 2. All conventional PCRs for this purpose were performed using QIAGEN Fast Cycling PCR Kit (QIAGEN) with varying conditions, as indicated in Table 2.

### 2.6. Sequencing and Phylogenetic Analyses

Conventional (RT-)PCR products were examined by automatic gel electrophoresis on QIAxcel Advanced System (QIAGEN). Before sequencing, most amplicons were purified using PCR Kleen Spin Columns (Bio-Rad, Hercules, CA, USA), according to the manufacturer’s instructions. Nucleotide sequences were obtained by Sanger sequencing using Mix2Seq Kits (Eurofins Genomics, Ebersberg, Germany), and identified by BLAST search (https://blast.ncbi.nlm.nih.gov/Blast.cgi, accessed on 22 June 2022).

The phylogenetic trees were constructed based on a short fragment that was created by a nested PCR using degenerate primers targeting a conserved region of herpesviral DNA polymerase genes (Table 1), and a 239-bp fragment of the *cytochrome b* gene (*cytb)*, with the respective primers located at positions 250 and 488 of *cytb* [41,43] (Table 2). In total, 41 sequences were generated: 26 herpesvirus and 15 rodent sequences. ClustalW multiple sequence alignments were conducted using BioEdit Alignment Editor Version 7.0.9.0. The phylogenetic trees were created in MEGA X [45], applying the neighbor-joining and p-distance methods with 1000 bootstrap replicates.

## 3. Results

### 3.1. Patho-Histological Results

Pathological investigation of the mouse carcasses revealed many parasites in the digestive tracts in three of them, non-suppurative myocarditis in one, central lobular necrosis in one liver, one myocardial fibrosis, a non-suppurative interstitial pancreatitis, and a nematode in the skeletal muscle of one house mouse.

### 3.2. Viral Nucleic Acid Identifications

All samples that were investigated, including oro-pharyngeal swabs, feces, organs, and ticks, were negative for all the viruses that were tested, except herpesviruses. In particular, 19 of the fecal and oro-pharyngeal swab samples from gerbils (35.8%), as well as seven of the murine organ samples (70.0%) were positive, while the Arabian spiny mouse was negative in the herpes consensus nested PCR. The resulting sequences were only partly identical to sequences in the GenBank database.

### 3.3. Sequencing and Phylogenetic Analyses

**Figure 1:**
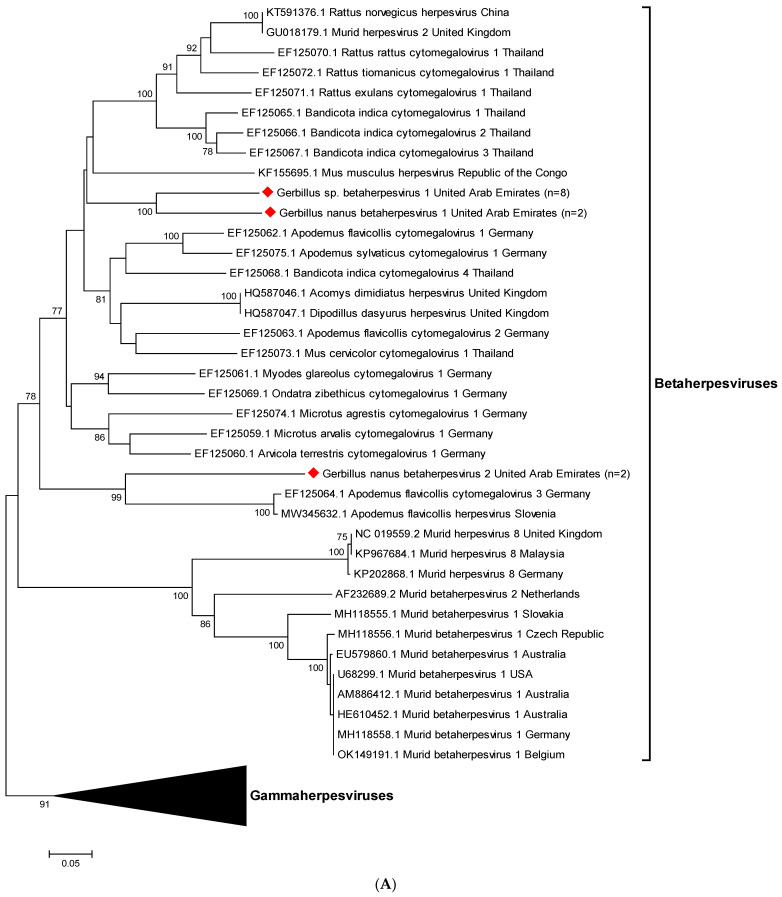

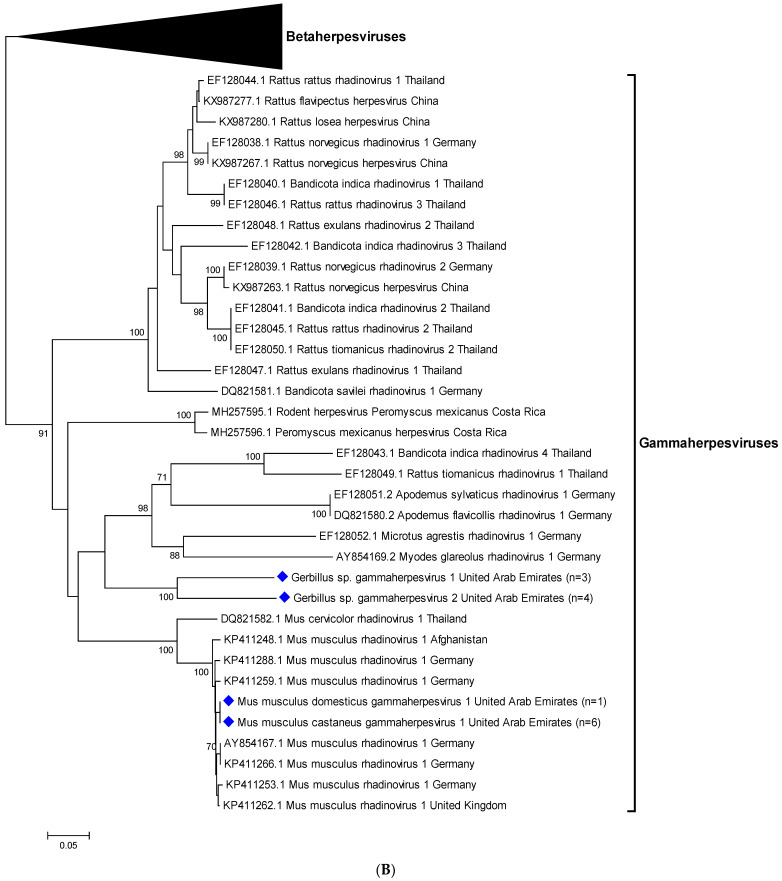
Phylogenetic tree of 74 partial beta- and gammaherpesviral DNA polymerase gene sequences. For each sequence, the corresponding GenBank accession number (reference sequences), host species and country of origin are indicated. Horizontal lines represent the genetic distances according to the scale at the bottom. Bootstrap values ≥70% are displayed at the nodes. (**A**) Expanded view of partial betaherpesviral DNA polymerase gene sequences that were detected in 12 oro-pharyngeal swab and fecal samples from gerbils. A total of eight identical sequences were detected in *Gerbillus* sp. samples and clustered together between *Rattus*, *Bandicota,* and *Apodemus* cytomegalovirus sequences. This novel strain was designated *Gerbillus* sp. *betaherpesvirus 1*. There were two identical sequences found in *Gerbillus nanus* samples that clustered next to this strain and were named *Gerbillus nanus betaherpesvirus 1*. Another two identical sequences found in *Gerbillus nanus* samples clustered further away, next to *Apodemus flavicollis* herpesviruses. This strain was named *Gerbillus nanus betaherpesvirus 2*. Sequences that were generated in this study are marked with red diamonds. (**B**) Expanded view of partial gammaherpesviral DNA polymerase gene sequences. Viral strains found in seven murine organ samples were 92–99% identical to *Mus musculus rhadinovirus 1* and were tentatively named *Mus musculus domesticus gammaherpesvirus 1* and *Mus musculus castaneus gammaherpesvirus 1*. Novel gammaherpesvirus strains that were detected in seven oro-pharyngeal swabs and fecal samples from gerbils clustered in two close, but separate branches, next to rhadinovirus 1 sequences from several different rodent species. We named them *Gerbillus* sp. *gammaherpesvirus 1* and *2*, respectively. Sequences that were generated in this study are marked with blue diamonds.

Analysis of the 12 partial betaherpesvirus sequences that were generated from oro-pharyngeal swab and fecal samples from gerbils resulted in three novel sequences (see Figure 1A). A total of eight of these sequences detected in *Gerbillus* sp. samples were identical and clustered together between *Rattus*, *Bandicota,* and *Apodemus* cytomegalovirus sequences. We preliminarily designated this novel strain *Gerbillus* sp. *betaherpesvirus 1*. Two identical sequences that were found in *Gerbillus nanus* samples clustered next to this strain and were named *Gerbillus nanus betaherpesvirus 1*. Another two identical sequences found in *Gerbillus nanus* samples clustered further away, next to *Apodemus flavicollis* herpesviruses, and were designated *Gerbillus nanus betaherpesvirus 2*.

Of the 14 partial gammaherpesvirus sequences that were established, seven were generated from gerbils and seven from house mice, resulting in four distinct novel sequences (see Figure 1B). Phylogenetic analysis revealed that the viral strains found in the seven murine organ samples were 92–99% identical to *Mus musculus rhadinovirus 1*. We named these two strains clustering together *Mus musculus domesticus gammaherpesvirus 1* and *Mus musculus castaneus gammaherpesvirus 1*, after the subspecies they were found in. The novel partial gammaherpesvirus sequences from seven gerbil samples clustered in two close, but separate branches, and were designated *Gerbillus* sp. *Gammaherpesvirus 1* and *2*, respectively. They are most closely related to rhadinovirus 1 sequences from several different rodent species.

### 3.4. Molecular Rodent Species Identification

**Figure 2:**
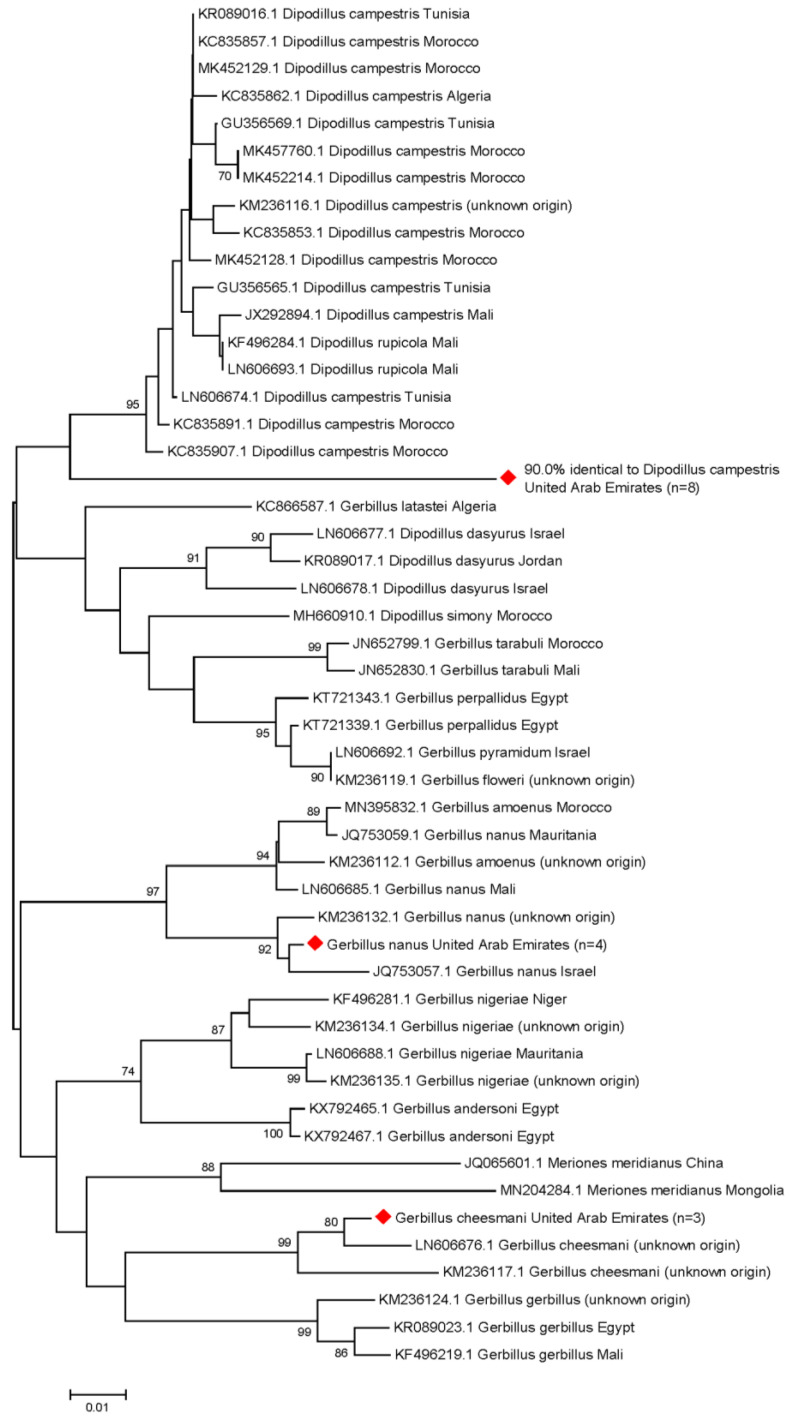
Phylogenetic tree of partial genomic sequences of *Gerbillus*, *Dipodillus* and *Meriones* species. Sequences that were generated in this study are indicated by red diamonds and represent groups of several individuals. Four of the herpesvirus-positive gerbils were identified as *Gerbillus nanus*, and three as *Gerbillus cheesmani*. Eight individuals clustered together in a separate clade, 90.0% identical to *Dipodillus campestris*. The sequences of the remaining four individuals were not analyzable. Phylogenetic analysis was performed on a 239-bp fragment within the *cytb* gene. For each reference sequence, the corresponding GenBank accession number, species name and country of origin are indicated (where known). Horizontal lines represent the genetic distances according to the scale at the bottom. Bootstrap values ≥70% are displayed at the nodes.

To verify the species of the herpesvirus-positive rodents that were sampled, we carried out several PCRs, the ones resulting in specific sequences are shown in Table 2. A total of six of the seven positive *Mus musculus* carcasses were identified as *Mus musculus castaneus*, and one as *Mus musculus domesticus*. From the 19 gerbil specimens, a short fragment of *cytb* was amplified and a phylogenetic tree was established (see Figure 2).

A total of four of the herpesvirus-positive gerbils were identified as *Gerbillus nanus*, and three as *Gerbillus cheesmani*. Interestingly, eight individuals clustered together in a separate clade, most closely related to the North African gerbil (*Dipodillus campestris*; 90.0%), five of which were positive for *Gerbillus* sp. *betaherpesvirus 1*, one for *Gerbillus* sp. *gammaherpesvirus 1*, and two for *Gerbillus* sp. *gammaherpesvirus 2*. The sequences of the remaining four individuals were not analyzable.

## 4. Discussion

As the majority of emerging infectious diseases originate from zoonotic reservoirs [32,33], rodents are a viable source of a broad spectrum of viruses. In this pilot study we focused on the rodent population in a desert reserve within the emirate of Dubai, comprised of various habitats such as gravel plains, sand dunes, and rock outcrops, which had been scarcely investigated, but represent only a small part of the rodent populations of the emirate. In our study, a larger sample size may have increased the likelihood of detecting active infections, and a metagenomics approach may have increased the number of rodent-specific virus taxa that were identified. However, our approach of screening for targeted viruses in this cohort, near known foci of zoonotic virus transmission, provides limited evidence to support the involvement of these rodents in the transmission of zoonotic viruses in the UAE.

From the typical members of the family Muridae found at the DDCR, we were able to sample several Cheesman’s gerbils, Balochistan gerbils, house mice, and according to morphological characterization, one Sundevall’s jird and one Arabian spiny mouse. In order to confirm the gerbil species identities, we amplified a fragment of *cytb*, because this gene is sufficiently variable and contains enough information to distinguish even closely related rodent species from each other [43]. However, the systematics of the genus *Gerbillus* is still heavily debated at several levels, with many species still awaiting confirmation of their taxonomic status, based on low numbers of specimens from locally limited areas [43]. Therefore, our attempts to confirm the identities of our gerbil specimens were partly impeded by the limited number of reference sequences available in this genomic region. Thus, eight individuals were assigned to a separate clade, 90.0% identical to the North African gerbil, which may either indicate the expansion of the geographic range of this gerbil species [46], or the existence of a closely related species in the UAE that has not yet been described. This unintentional result from our study highlights the importance of identifying zoonotic viruses in wildlife, as this not only informs public health but also contributes to understanding biodiversity in some under-represented geographic regions.

We assumed there was a high possibility to find MERS-CoV or CCHFV in the sampled rodents and ticks because dromedary camels were kept nearby that were mainly used for tourism purposes. By concurrent sampling of these camels, we found CCHFV- as well as MERS-CoV-reactive antibodies in almost all of them [7,47,48]. CCHFV is a tick-borne virus, primarily maintained in ticks of the *Hyalomma* genus, and several mammalian livestock species act as amplifying hosts [7,8,9]. Ticks are potent vectors for pathogens because of their blood-feeding behavior, and their wide variety of possible vertebrate hosts. Immature stages of ticks involved in the transmission of human pathogens often feed on birds and small mammals, and feed on larger mammals when adult. Thus, ticks link diverse branches of vertebrates and may collect and spread pathogens from a large variety of vertebrate hosts [49].

Since the exact transmission cycle of MERS-CoV and the animals involved have yet to be determined [50,51], it is also possible that rodent species are involved. It has been observed in closed camel herds that MERS-CoV infections persist for a certain period of time and then cease for a while, until suddenly cases rise again without the introduction of dromedaries from outside the herd. This may be due to seasonality effects based on the time of calving during winter months, and subsequent rises in prevalence once a critical number of susceptible young animals is reached [52]. However, another possible explanation for re-introduction events may be the presence of persistently infected rodents, which shed the virus with their secreta and excreta. Rodents have important roles in the transmission cycles of many pathogens. For instance, they have been involved in the evolution of several human coronaviruses, but their exact role in the transmission of MERS-CoV remains unclear [53]. Hence, we screened our samples for this virus as well, including the collected ticks, although MERS-CoV infection does not involve a significant viremic period, and it has recently been shown that apparently neither ticks nor rodents play a role in the transmission cycle [53,54]. Concordant with the findings of Hemida et al., but in contrast to our findings with CCHFV [7,9], in this cohort we could not find any indications for the involvement of rodents or rodent-associated ticks in the transmission of CCHFV or MERS-CoV.

Hantaviruses are mostly rodent-borne viruses with significant zoonotic potential that are carried by peri-domestic as well as wild rodents worldwide. Human infection can result in substantial morbidity and mortality. There are two categories: Old World and New World orthohantaviruses, the former being transmitted by rodents of the Arvicolinae and Murinae subfamilies found across Europe and Asia, the latter are carried by rodents of the Sigmodontinae and Arvicolinae subfamilies from the North and South American continents. The notable exception is the brown rat (*Rattus norvegicus*), which is the natural host for Seoul virus, being distributed worldwide due to international trade [13]. Another typically rodent-borne virus is LCMV, with its primary host being the house mouse, but it has also been found in other rodents, and many other mammals have been experimentally infected, such as rabbits, dogs, and pigs [15,55,56]. RusV, the causative agent of ‘staggering disease’ in domestic cats, was first identified in zoo animals, and later also in yellow-necked field mice in Germany. Recently, we were able to confirm the presence of RusV in samples of cats and wood mice [17]. Whether rodents are the primary source of infection remains to be further investigated; here, we could not find any of these viruses in rodents of the UAE.

Flaviviruses are usually transmitted in enzootic cycles between birds and bird-biting mosquitoes, especially of the *Culex* genus, or by ticks, which feed on small mammals including rodents. Humans become infected incidentally via bites from the arthropod vectors, but are considered dead-end hosts, as they normally do not develop sufficiently high or lasting viremia to pass the virus on to other vectors [26,57]. Some mosquito-borne flaviviruses, for example WNV, have been found in ticks [26,57], and gerbils can be experimentally infected with both mosquito- and tickborne flaviviruses [58,59]. Although the flavivirus AHFV has mainly been found in Saudi Arabia, since 2010 there is evidence of a wider distribution, with four infected Italian tourists returning from Egypt, and two studies from Djibouti that found neutralizing antibodies to AHFV in human serum, and two positive ticks that were collected from cattle [10]. However, from our data, we cannot report its presence in rodents or ticks in the UAE, nor the presence of any other flaviviruses that were tested. This may be due to the rather small sample size, which is the main limitation of this study.

Herpesviruses have been detected in a vast variety of species, each strain closely associated with its respective primary host. Upon infection of other organisms, severe or even lethal diseases may occur, predominantly in the case of alphaherpesviruses, which are characterized by a short replication cycle, destruction of infected cells, and the establishment of latency in sensory ganglia [31]. However, alphaherpesviruses that naturally infect Rodentia species are currently unknown [60]. In the present study, we characterized herpesvirus sequences from 19 gerbils (35.8%) and seven mice (70.0%) that were only partly identical to sequences in GenBank. In total, we detected three novel partial betaherpesvirus sequences in gerbils, and four novel partial gammaherpesvirus sequences in gerbils and house mice. The strains that were found in mice were 92–99% identical to *Mus musculus rhadinovirus 1*, which was the first gammaherpesvirus of *Mus musculus* discovered in Germany and the UK in 2007 [60]. The other strains that were found in gerbils could not be assigned to any existing branches of the herpesvirus phylogenetic tree and were thus deemed novel strains. Each of the novel herpesviruses described here, and their further characterization in the future, may improve our understanding of herpesvirus biology.

## 5. Conclusions

In this pilot study, we could not detect any of the potentially zoonotic viruses in the sampled rodents and attached ticks. However, we identified three novel partial betaherpesvirus sequences in gerbils, tentatively designated *Gerbillus* sp. *betaherpesvirus 1*, and *Gerbillus nanus betaherpesvirus 1* and *2*, as well as four novel partial gammaherpesvirus sequences in gerbils and house mice, named *Gerbillus* sp. *gammaherpesvirus 1* and *2*, *Mus musculus domesticus gammaherpesvirus 1*, and *Mus musculus castaneus gammaherpesvirus 1*. Interestingly, phylogenetic analysis revealed that eight gerbils were most closely related to *Dipodillus campestris*, the North African gerbil (90.0%), which either indicates the expansion of the geographic range of this species [46], or the existence of a closely related species in the UAE, which has not yet been described.

## Figures and Tables

**Table 1:** PCR conditions, primers, and probes that were used for the detection of viruses.

Virus (Family)	Primer/Probe Name	Sequence (5′-3′)	Amplicon Length	PCR Kit/Conditions		Reference
MERS-CoV (*Coronaviridae*)	Orf1a-F Orf1a-R Orf1a-P	CCACTACTCCCATTTCGTCAG CAGTATGTGTAGTGCGCATATAAGCA TTGCAAATTGGCTTGCCCCCACT	83 bp	Quantabio qScript XLT 1-Step RT-qPCR ToughMix		RT-qPCR; Corman et al. [37]
CCHFV (*Nairoviridae*)	-	Data not provided by manufacturer	-	50 °C for 10 min; 95 °C for 2 min; 45x 95 °C for 15 s, 55 °C for 45 s, and 72 °C for 15 s		RealStar RT-PCR Kit 1.0; altona Diagnostics
AHFV (*Flaviviridae*)	NS3-5439F NS3-5565R NS3-5474P	CAGGGGAGACAGAATTGGGAAG TCATGAGCACCAAAGCGCAC TGAAGCCCATTGGACCGATCCACATAGCAT	84 bp	Quantabio qScript XLT 1-Step RT-qPCR ToughMix		RT-qPCR; in-house method
Hantaviruses (*Hantaviridae*)	HAN-L-F1 HAN-L-R1	ATGTAYGTBAGTGCWGATGC AACCADTCWGTYCCRTCATC	452 bp	QIAGEN OneStep RT-PCR Kit		Nested RT-PCR; Klempa et al. [38]
	HAN-L-F2 HAN-L-R2	TGCWGATGCHACIAARTGGTC GCRTCRTCWGARTGRTGDGCAA	390 bp	QIAGEN OneStep RT-PCR Kit: 50 °C for 1 min; 95 °C for 1 min; 40x 94 °C for 30 s, 55 °C for 30 s and 72 °C for 30 s; 72 °C for 7 min		
LCMV (*Arenaviridae*)	LCM-S-F LCM-S-R	CTGTGAGYGCYTGCACAACATC GATCCTAGGCATTTGATTGCGC	650 bp	QIAGEN OneStep RT-PCR Kit		RT-PCR; in-house method
RusV (*Matonaviridae*)	RusV-234F RusV-323R RusV-256P	CCCCGTGTTCCTAGGCAC TCGCCCCATTCWACCCAATT GTGAGCGACCACCCAGCACTCCA	51 bp	Quantabio qScript XLT 1-Step RT-qPCR ToughMix		RT-qPCR; Matiasek et al. [17]
Poxviruses (*Poxviridae*)	Pan-Pox-F Pan-Pox-R	ACACCAAAAACTCATATAACTTCT CCTATTTTACTCCTTAGTAAATGAT	220 bp	QIAGEN Fast Cycling PCR Kit: 95 °C for 5 min; 50x 96 °C for 5 s, 50 °C for 5 s, 68 °C for 10 s; 68 °C for 1 min		Pan-Pox PCR (low GC); Li et al. [39]
Flaviviruses (*Flaviviridae*)	Flavi-S Flavi-AS2	TACAACATGATGGGGAARAGAGARAA GTGTCCCAGCCNGCKGTGTCATCWGC	260 bp	QIAGEN OneStep RT-PCR Kit		Universal RT-PCR; Patel et al. [40]
Herpesviruses (*Herpesviridae*)	HerpesCons-F1 HerpesCons-F2 HerpesCons-R	GAYTTYGCNAGYYTNTAYCC TCCTGGACAAGCAGCARNYSGCNMTNAA GTCTTGCTCACCAGNTCNACNCCYTT	700 bp	QIAGEN Fast Cycling PCR Kit: 95 °C for 5 min; 50x 96 °C for 5 s, 50 °C for 5 s, 68 °C for 25 s; 72 °C for 1 min		Nested PCR; VanDevanter et al. [41]
	HerpesCons-nF HerpesCons-nR	TGTAACTCGGTGTAYGGNTTYACNGGNGT CACAGAGTCCGTRTCNCCRTADAT	215–315 bp	QIAGEN Fast Cycling PCR Kit: 95 °C for 5 min; 50x 96 °C for 5 s, 50 °C for 5 s, 68 °C for 8 s; 72 °C for 1 min		

**Table 2:** PCR conditions and primers that were used for rodent species identification.

Primer Name	Sequence (5′-3′)	Amplicon Length	PCR Conditions	Reference
L2513-F H2714-R	GCCTGTTTACCAAAAACATCAC CTCCATAGGGTCTTCTCGTCTT	243 bp	95 °C for 5 min; 50x 96 °C for 5 s, 57 °C for 5 s, 68 °C for 15 s; 68 °C for 1 min	Kitano et al. [42]
GERBCYTB-F2 GERBCYTB-R3	GCAAACGGAGCCTCAATATT CATTCTACRATTGTTGGGCCA	239 bp	95 °C for 5 min; 50x 96 °C for 5 s, 55 °C for 5 s, 68 °C for 30 s; 72 °C for 1 min	Ndiaye et al. [43]
VF1d VR1d	TGTAAAACGACGGCCAGTTYT-CNACHAAYCAYAAAGAYATYGG CAGGAAACAGCTATGACTANA-CYTCNGGRTGNCCRAARAATCA	659 bp	95 °C for 2 min; 5x 96 °C for 10 s, 50 °C for 10 s, 68 °C for 30 s; 35x 96 °C for 10 s, 54 °C for 10 s, 68 °C for 30 s; 72 °C for 1 min	Modified from Ivanova et al. [44]

## Data Availability

The sequences that were determined in this study were too short to be submitted to GenBank database, however the corresponding author is happy to share them or any other raw data of this study with colleagues on request.
